# Asymmetries of Influence: Differential Effects of Body Postures on Perceptions of Emotional Facial Expressions

**DOI:** 10.1371/journal.pone.0073605

**Published:** 2013-09-10

**Authors:** Catherine J. Mondloch, Nicole L. Nelson, Matthew Horner

**Affiliations:** Department of Psychology, Brock University, St. Catharine’s, Ontario, Canada; University of Tuebingen Medical School, Germany

## Abstract

The accuracy and speed with which emotional facial expressions are identified is influenced by body postures. Two influential models predict that these congruency effects will be largest when the emotion displayed in the face is similar to that displayed in the body: the emotional seed model and the dimensional model. These models differ in whether similarity is based on physical characteristics or underlying dimensions of valence and arousal. Using a 3-alternative forced-choice task in which stimuli were presented briefly (Exp 1a) or for an unlimited time (Exp 1b) we provide evidence that congruency effects are more complex than either model predicts; the effects are asymmetrical and cannot be accounted for by similarity alone. Fearful postures are especially influential when paired with facial expressions, but not when presented in a flanker task (Exp 2). We suggest refinements to each model that may account for our results and suggest that additional studies be conducted prior to drawing strong theoretical conclusions.

## Introduction

Emotional expressions provide important cues about the environment and facilitate appropriate approach or avoidance behavior [1]. People can detect the emotional status of other individuals using a variety of cues including facial expression [2,3], body posture [4–8], body movements (even in a point-light display [9]) and, to some extent, vocal intonation (e.g., [10–12]). It is surprising, then, that theories of emotion perception have largely been built on studies where adults labeled or categorized isolated faces (as discussed in 13–15). This practice reflects the influence of the discrete category view of emotion perception, which assumes that discrete emotions are perceived categorically [16–18], with some facial expressions being recognized universally and with ease: happy, sad, anger, fear, disgust, surprise. In contrast, during daily social interactions, faces are encountered in contexts that include a wealth of cues to emotion (e.g., body postures, objects, voices, scenes). Recent studies provide compelling evidence that these cues influence adults’ perception of emotion and, most notably, can alter their perception of facial expressions.

Although adults are sensitive to emotion cues presented in isolation, their ability to accurately determine emotional status is enhanced when multiple cues are available; Aviezer and colleagues [19] found that participants’ accuracy for isolated disgust faces was only 65%, but rose to 91% when a disgust face was paired with a disgust body and a dirty diaper. Conversely, participants’ accuracy in recognizing facial displays of emotion is impaired when the facial display is incongruent with the other cues present. For example, adults’ accuracy decreases and response times increase when asked to make a two-alternative forced-choice judgment about a fearful face presented on a body posing anger relative to when the face is presented on a body posing fear (first shown in [20]).

Matsumoto and Hwang [21] question the validity of asking participants to categorize facial expressions paired with incongruent bodies. They claim that having the “technological capability to mix and match many different types of stimuli for judgments …..does not necessarily mean that (doing so) has relevance to the real world” (p. 399). However, as noted by Hassin, Aviezer, and Bentin [22], the frequency with which incongruent stimuli are encountered in daily life is unknown (and certainly no less frequent than encountering isolated faces); there are many conditions under which mis-matched facial expressions and body postures are expected (e.g., when people attempt to regulate or mask their emotions). Furthermore a complete absence of incongruent stimuli in the real world would not diminish the utility of this approach in understanding emotion perception. We note that it is not unlike the approach taken by Konrad Lorenz and Niko Tinbergen, who unraveled many mysteries of animal behaviour by presenting animals with supernormal stimuli, stimuli that animals would never encounter in their daily environment: a thin red rod marked with white bands (to herring gull chicks [23]), crude models of conspecifics with and without red bellies (to stickleback fish [24]), or enormous or even cube-shaped eggs (to greylag geese [25]). It is the use of our technological capabilities to create incongruent stimuli that has highlighted the existence of congruency effects, which provide compelling evidence that the hypothesized one-to-one mapping of facial expressions to perceived emotion category is surprisingly fragile.

Broadly, the presence of conflicting emotion cues interferes with the emotion perceived. Body posture and background scenes influence adults’ reaction times and the expression perceived in faces [13,14,19,26–29]. Likewise, tone of voice and hand gestures both influence adults’ perception of facial expressions [30,31], body posture influences adults’ perception of emotional voices [32], and the emotional content of music influences adults’ perception of body movements [33]. Finally, situational cues in the form of short vignettes can influence adults’ labeling of a subsequently presented face [34]: a sad face was labeled as sad when presented in isolation but was labeled ‘disgust’ when preceded by a disgust-related vignette.

Two influential models of emotion are consistent with these congruency effects (decreased accuracy and increased response times associated with incongruent contexts), although they make some divergent predictions about the relative magnitude of effects across emotion pairings and offer different, though perhaps complimentary, explanations. According to the dimensional (or circumplex) theory of emotion perception [35–38], people directly perceive valence (pleasant vs. unpleasant) and arousal (low vs. high) and subsequently infer the underlying emotion. For example, when a face is negative in valence and low in arousal we perceive sadness. Therefore, congruency effects should be maximal when the two emotions are similar on these two underlying dimensions because under these conditions the precise emotion being displayed is difficult to infer (see 34). Dimensional theories predict that congruency effects will be maximal, but not limited to, conditions in which one source (e.g., the face) displays anger and the other source (e.g., body posture) displays fear because both of these emotions are negatively valenced and high in arousal. Congruency effects will be smaller for emotions that differ on only one dimension, such as arousal (e.g., sad and fear), and negligible/absent for emotions that are opposite on both arousal and valence (e.g., sad and happy). Consistent with the dimensional theory, congruency effects may arise because contextual cues alter emotion perception by directly altering the perceived valence and arousal of emotional faces [19]: Disgust faces paired with pride bodies and props were rated more positively than disgust faces paired with disgust bodies and props, and sad faces paired with fearful bodies and props were rated as higher in arousal than sad faces paired with sad bodies and props.

According to the emotional seed model [13], a more recent characterization of emotion perception, adults’ perception of emotions depends on the physical characteristics of the expression (e.g., raised brows, wrinkled nose). Some physical characteristics are diagnostic of only one emotion, such as the wrinkled nose in disgust faces, whereas other characteristics are shared among multiple emotions. These shared characteristics are reflected in adults’ similarity judgments and classifications made by computer models [39]. For example, anger and disgust share furrowed brows, fear and surprise share raised brows and wide eyes, and fear and sad share oblique eyebrows pulled together [40,41]. The emotional seed model predicts that congruency effects will occur when the number of shared physical characteristics (what Aviezer, Hassin, Bentin, et al. [13], dub *emotional seeds*) in the facial displays associated with each emotional source is high. Support for the emotional seed model comes from evidence that the magnitude of congruency effects is directly related to physical similarity [19]: Categorization of faces displaying disgust was most impaired when paired with bodies posing anger (the most similar facial expression), was least impaired when paired with bodies posing fear (the least similar expression), and was moderately impaired when paired with bodies posing sadness.

However, we note that predictions made by these two models do not always conflict. Evidence that congruency effects are found when participants are judging fear and sad expressions (two expressions that vary on only one dimension and that share physical characteristics), but absent for sad versus happy expressions (two expressions that differ on two dimensions and that share minimal characteristics) [42] is consistent with predictions made based on both the dimensional theory and the emotional seed model.

Congruency effects have only recently been implemented as a tool to investigate emotion perception. Aviezer, Hassin, Bentin et al. [13] provide a compelling argument that congruency effects provide strong support for the emotional seed model. However, the two-alternative forced-choice tasks used in the vast majority of studies investigating congruency effects (e.g., [20,27,28,42]) do not allow for multiple emotion comparisons (e.g. anger vs. sad and sad vs. fear) and therefore are not an ideal tool for investigating the relative influence of physical versus dimensional similarity. We argue that prior to drawing strong theoretical conclusions linking congruency effects to models of emotion perception, a richer data set is needed – one that includes data collected using a variety of methods.

In a recent study by Aviezer, Trope, and Todorov [26], adults were presented with four different emotions conveyed by faces and bodies (anger, fear, sad, and disgust). Aviezer et al. [26] found that congruency effects were smaller when faces and bodies were misaligned, suggesting that congruency effects reflected holistic processing (see also 42). They did not attempt to explain their results using the dimensional theory vs. emotional seed model but their findings suggest that the pattern of congruency effects is likely to be complex. Most notably, they reported asymmetries in congruency effects. Sad bodies interfered with adults’ perception of angry faces but angry bodies did not interfere with adults’ perception of sad faces; likewise, fear bodies interfered with adults’ perception of sad faces more than sad bodies interfered with adults’ perception of fear faces. Similarly, in a study by Van den Stock, Righart, and de Gelder [32], happy bodies interfered with adults’ perception of fearful faces, but fearful bodies did not interfere with their perception of happy faces. Such asymmetries indicate that similarity per se cannot entirely explain congruency effects; after all, sad faces are as similar to fear faces as fear faces are to sad faces! Rather, asymmetries suggest that some facial expressions may be especially vulnerable to congruency effects and that some body postures may be especially influential. One important factor, for example, may be saliency. Threatening (angry) faces pop out of a crowd of neutral or happy faces [43], suggesting that angry faces may be more salient and less vulnerable to congruency effects than other emotions. Not all studies allow for an examination of asymmetries. Aviezer, Hassin, Ryan, et al [19] asked participants to choose from four emotions, but the only facial expression presented was disgust; participants may have been hesitant to label every expression as disgust, thus enhancing congruency effects by encouraging participants to look to contextual cues to make their judgments. Furthermore, asymmetries in congruency effects could not be revealed.

The purpose of the current study was to expand evidence for the nature of congruency effects such that participants were asked to make 3-alternative forced-choice judgments for three negatively valenced emotions: sad, anger, and fear. Each facial expression (e.g., sad) was presented on congruent (i.e., sad) and incongruent (i.e., anger, fear) postures. Doing so allowed us to examine whether the magnitude of congruency effects varied mainly as a function of facial expression (with some facial expressions being more vulnerable than others), body postures (with some postures being more influential than others), or specific combinations of facial expressions and body postures, perhaps based on similarity (see 13).

**Figure 1:**
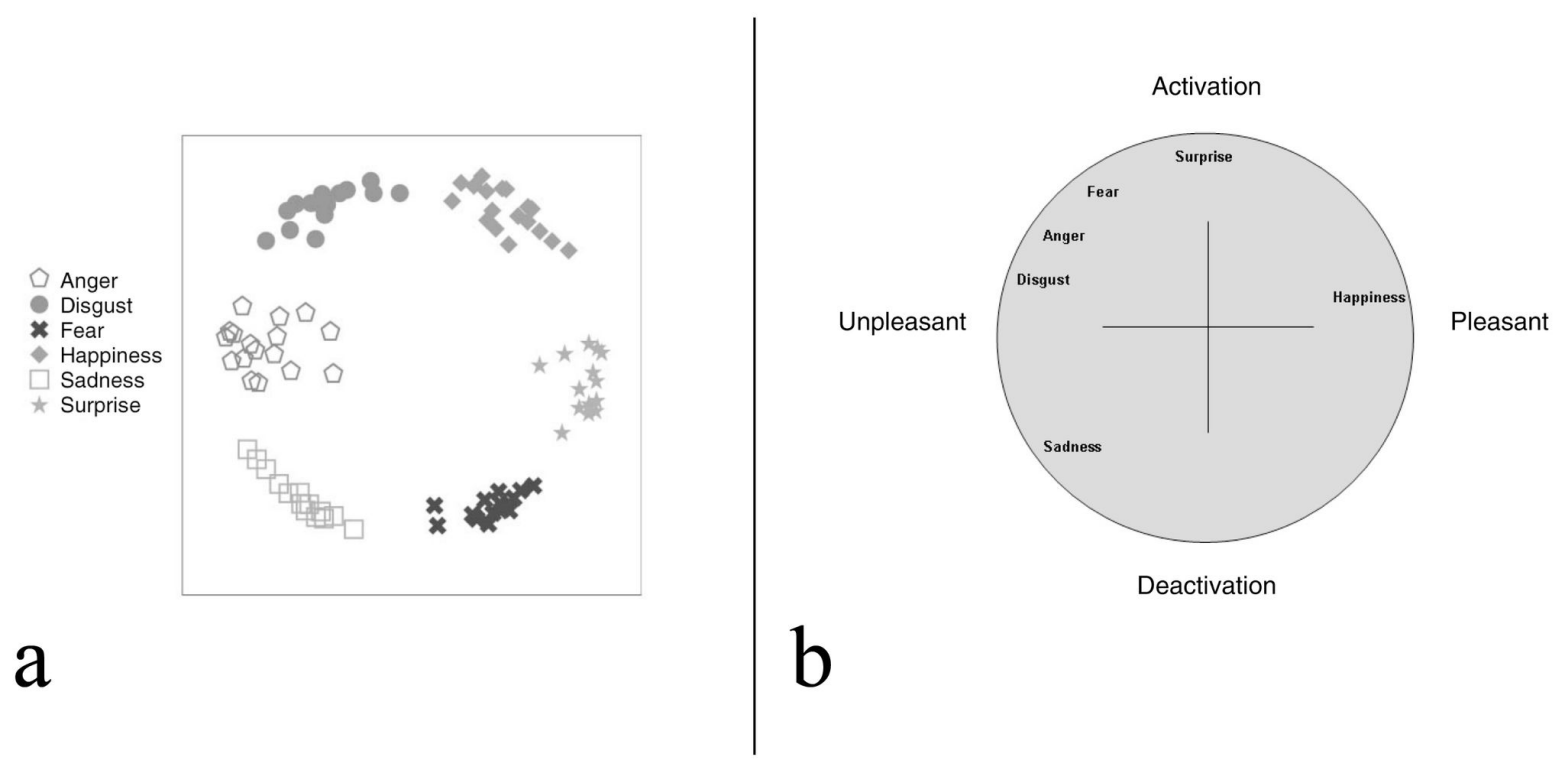
Models of emotion perception. The relative position of emotions as organized by (a) the Seed Model [13] and (b) the Dimensional model [3]. Reprinted from Aviezer, Hassin, Bentin, & Trope, 2008, Putting Facial Expressions Back in Context [13]. Copyright Guilford Press. Reprinted with permission of The Guilford Press.

Furthermore, these emotions were selected because the two models predict different patterns of congruency effects. Although both models predict larger congruency effects when the emotion displayed in the face is similar to that displayed in the context, the two models differ on their characterization of similarity. The dimensional theory emphasizes similarity in valence and arousal whereas the emotional seed model emphasizes similarity in physical characteristics of facial expressions. According to the emotional seed model (Figure 1a) sad faces are more similar to both angry and fearful faces than angry and fearful faces are to each other. Consequently, this model predicts larger congruency effects when sadness is present in the face or body and paired with fearful or angry stimuli than when fearful and angry stimuli are paired with each other. In contrast, according to the dimensional theory (Figure 1b) fear and anger are more similar to each other than either is to sadness; although all three emotions are negatively valenced, only fear and anger are high in arousal. Consequently, this theory predicts smaller congruency effects when sadness is present in the face or body and paired with fearful or angry stimuli than when fearful and angry stimuli are paired with each other.

We measured congruency effects in three different ways: proportion of correct responses, median response times on correct trials, and biases in the pattern of errors observed (i.e., whether the proportion of ‘fear’ errors made in response to sad faces increased when sad faces were presented on fearful bodies). To ensure that our results were driven by the expressions presented, rather than props (e.g. a hand holding a gun), as has been used in prior research [13,19,26], we presented faces on congruent and incongruent body postures but excluded all other contextual cues. Previous studies investigating congruency effects have used a range of presentation times and so in Experiment 1a, incongruent face/body pairs were presented briefly and in Experiment 1b the same stimuli were presented for an unlimited time. In Experiment 2 we used a flanker task to determine the accuracy with which participants could classify target postures and whether some postures produced greater interference than others, even when faces were absent.

## Experiment 1a

### Introduction

In Experiment 1a, we showed adults sad, fearful, and angry faces presented on both congruent and incongruent bodies to investigate the magnitude of congruency effects across facial expressions, body postures, and specific face-body pairings with specific attention to asymmetries in congruency effects. A second goal was to contrast predictions made by the dimensional theory (larger effects when face-body stimuli display combinations of fear and anger) versus the emotional seed model (larger effects when either the face or body displays sad).

### Method

#### Participants

Undergraduate students (*n* = 24) between the ages of 19 and 25 (*M* = 21.6) participated; all had normal/corrected-to-normal visual acuity and received partial course credit or a small monetary gift. One additional participant was excluded from analysis because he failed criterion trials (see procedure for more details).

#### Materials

Facial expressions were taken from the NimStim Face Stimulus Set [44]; the emotion displayed by each image was correctly identified by over 70% of participants in a validation study [44]. Six models (three male) each posed a sad, angry, and fearful facial expression. Four of these models (two male) also posed a happy expression. All face images were resized to approximately 2.2 cm horizontally x 2.8 cm vertically and cropped such that each model’s hair and face contour were similar across expressions. Body postures were taken from a set of stimuli created in our lab (see 42 for validation details). Four different models (two male) each provided two sad, two angry, and two fearful postures for test trials. Each model also provided one happy body posture that was presented during catch trials. All face and body models used in this study provided consent for publication of the photos in publications and on the web.

**Figure 2:**
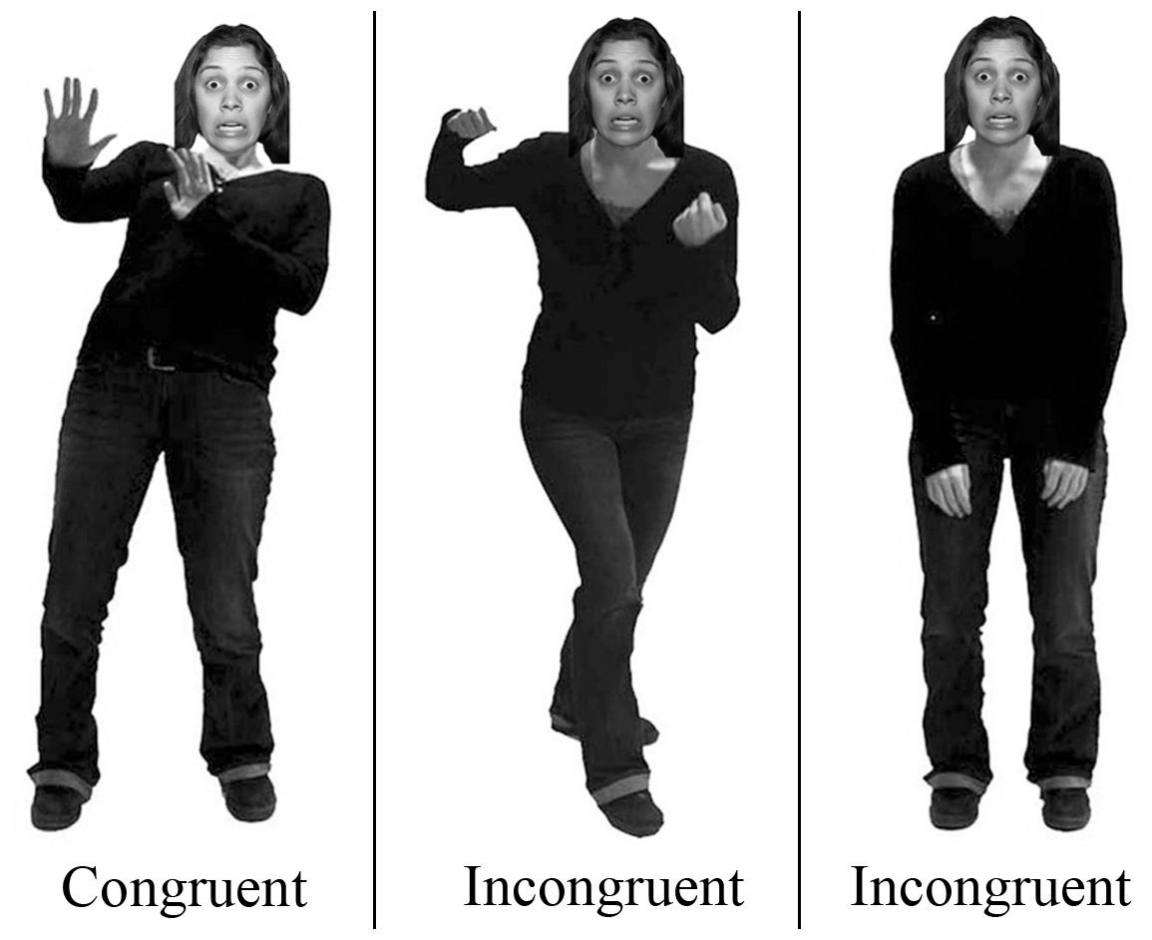
Incongruent and congruent stimuli. Examples of Congruent (fearful face on fear body) and Incongruent (fearful face on angry body; fearful face on sad body) stimuli from Experiments 1a and 1b. All expressions used in these studies were obtained from the NimStim face set [44] and all models provided consent for publication of the photos in publications and on the web. Models for body expressions have given written informed consent, as outlined in the PLOS consent form, to publication of their photograph.

Test stimuli were created by aligning each face with several postures posed by a same-sex model (Figure 2). Congruent stimuli (e.g., sad face on a sad body) were created by aligning each negatively-valenced face stimulus (sad, fearful, and angry) with four matching body postures (two same sex models x two postures). Incongruent stimuli (e.g., sad face on an angry body) were created by aligning each face with eight non-matching body postures (two incongruent expressions x two same sex models x two postures). Stimuli for catch trials were created by aligning each happy face with one body posture of each of the four emotions. Thus, there were 216 different test stimuli (72 congruent) and 16 catch stimuli. All stimuli were realistically proportioned creating a face to body ratio of approximately 1:6 (see 20). The task was programmed with Cedrus Superlab Version 4 and participants were required to make their responses with a Logitech controller.

#### Procedure

All work was conducted according to the principles expressed in the Declaration of Helsinki. Experiments 1a, 1b, and 2 received clearance from the Brock University Research Ethics Board, 04-035. Each participant provided informed written consent. After providing written consent and completing our visual screening procedure, participants were seated in front of a 23-inch LG computer monitor at a distance of 60cm. The entire protocol was comprised of three blocks of trials that were presented in a fixed order: isolated faces, test trials, and isolated bodies. In each trial throughout the protocol, a central fixation stimulus (*), positioned in the shoulder region of test stimuli, was presented for 1000ms; this was followed by a test stimulus and then a blank screen during which the participant responded by pressing one of three buttons on the controller.

The isolated faces block was designed to ensure that each participant accurately identified angry, sad, and fearful faces in the absence of context. During each of six practice trials, isolated faces (two per emotion; half male) were presented for 2000ms; during each of 12 criterion trials (four per emotion; half male) stimuli were presented for 600ms. We presented stimuli in this and subsequent phases for 600ms to be consistent with the method used in our previous study in which we investigated congruency effects in children and adults [42]. Participants were instructed to press the button corresponding to the emotion depicted in the face and to do so as quickly as possible without making errors; buttons were labeled ‘F’ (fear), ‘S’ (sad), and ‘A’ (anger). Participants were required to respond correctly on 10 of the 12 criterion trials to be included in the study; each participant was allowed three attempts to do so. Only one adult failed to meet this criterion; accuracy for participants who were included in the analyses was very high (*M* = .91).

Prior to test trials, participants were instructed to indicate which of the three emotions was displayed in the face and to ignore the body. Twelve practice trials were administered during which stimuli were presented for 2000ms (4 trials) and then for 600 ms (8 trials). Participants then completed 216 test trials (72 congruent; 144 incongruent) that were presented in a different random order to each participant. Sixteen catch trials, in which happy faces were presented on happy (*n* = 4) or negatively valenced (*n* = 12) body postures, were embedded amongst the test trials. Participants were instructed to withhold from pressing any button when they detected a happy face and to simply respond verbally by saying ‘happy’. Catch trials served to verify that participants were attending to the face; participants were required to respond correctly on at least 13 catch trials to be included in the analysis. Every adult met this criterion (mean proportion correct = .99).

The isolated bodies block was designed to verify that each participant could correctly identify the emotion conveyed by body postures. Each of the 24 body postures was presented once for 600ms; heads were visible, but the faces were blurred. Participants were required to accurately identify 20 of the 24 emotional body postures to be included in the final analysis because failure to recognize the emotional body postures would render any congruency effects impossible to interpret. Every participant met this criterion (mean proportion correct = .97).

### Results

#### Analyses

Accuracy and median response times on correct trials were analyzed with a 3 (face emotion: sad, fear, anger) x 3 (body emotion: sad, fear, anger) repeated-measures ANOVA. We followed up significant interactions with one-way repeated-measures ANOVAs in which the influence of body posture was tested separately for each facial expression. Two-tailed paired samples t-tests planned a priori were conducted to determine whether accuracy differed between congruent and incongruent trials and whether accuracy differed across the two incongruent trials (e.g., sad faces on fearful versus angry bodies).

In addition, we investigated the influence of body posture on the pattern of errors made for each facial expression to determine whether incongruent body postures altered the pattern of errors made. For any face stimulus (e.g., sad) an incorrect response can take two forms (e.g., fear or anger) and the proportion of errors that fall into these two categories are not independent. Therefore, for each facial expression, we arbitrarily selected one of the two possible errors as the target error (e.g., fear on sad face trials) and analyzed the proportion of that target error across congruent (sad bodies) and the two types of incongruent (fear bodies, angry bodies) trials. For congruent stimuli, we conducted three one-sample t-tests (one per facial expression) comparing the proportion of target errors to .5, to determine whether a response bias was present even when facial and postural emotions were congruent. For incongruent stimuli, we conducted three one-way repeated-measures ANOVAs (one per facial expression) in which the proportion of target errors was compared across the three body postures. Simple contrasts were conducted to follow-up on significant ANOVAs. A shift in bias would be evident if the proportion of target errors increased, as compared to congruent trials, when the body matched the target (e.g., when sad faces were shown on fearful bodies) and decreased when the body did not match the target (e.g., when sad faces were shown on angry bodies).

#### Accuracy

**Figure 3:**
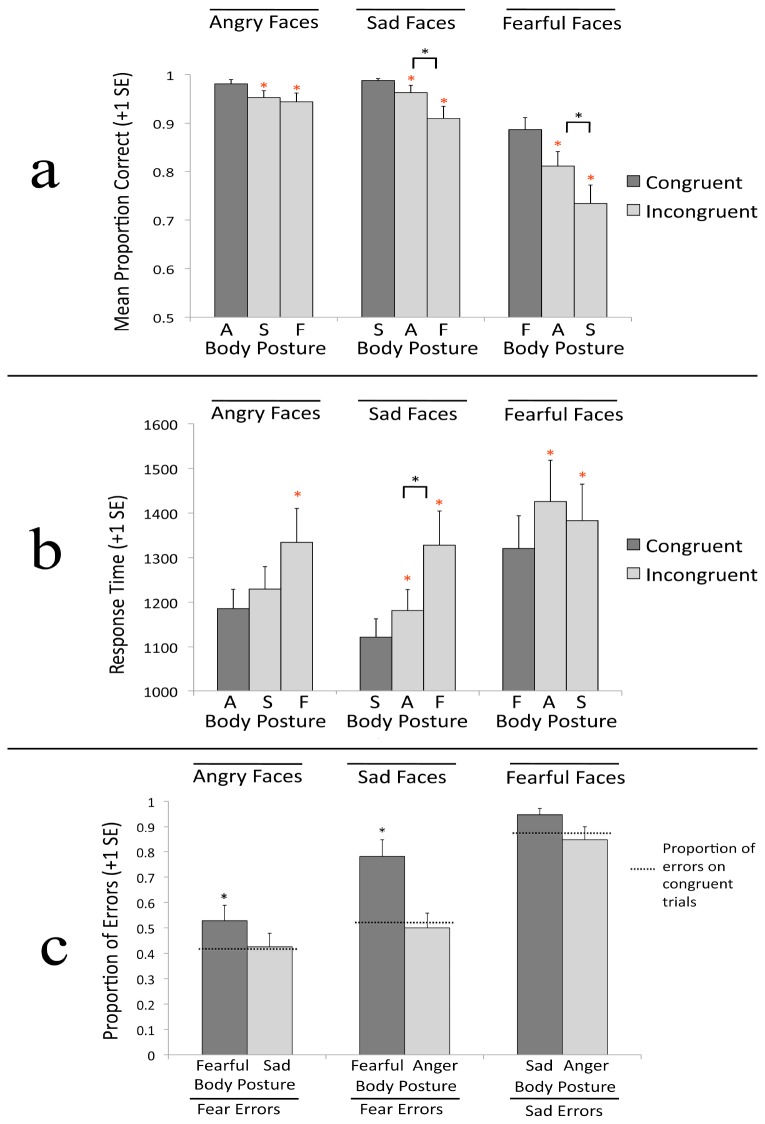
Results for Experiment 1a. a) Proportion of correct responses for each facial expression, presented by posture. b) Median reaction time results for each facial expression, presented by posture. Results are presented in milliseconds. c) Proportion of errors made for each facial expression, presented by posture. Dotted line represents the proportion of errors made by participants viewing congruent expressions, and was used as a baseline error level. * over individual bars indicate a significant difference between congruent and incongruent trials.

The main effect for face emotion was significant, *F*(2,46) = 27.716, *p* < .001, η^2^ = .546. As shown in Figure 3a, accuracy was lower for fearful faces (*M* = .73) than for angry faces (*M* = .91), *t*(23) = -6.951, *p* < .001, d = 1.4, and sad faces (*M* = .89), *t*(23) = -4.927, *p* < .001, d = 1.01. Accuracy did not differ for sad versus angry faces, *p* = .326. The main effect for body emotion was also significant, *F*(2,46) = 3.911, *p* = .027, η^2^ = .145. Accuracy was lower for facial expressions paired with sad bodies (*M* = .83) than for facial expressions paired with angry bodies (*M* = .86), *t*(23) = -3.065, *p* = .005, d = .63, but not fearful bodies (*M* = .83), *t*(23) = -.363, *p* = .72. The difference in accuracy for facial expressions paired with fearful versus angry bodies was not significant, *p* = .078. These main effects were qualified by a significant face emotion x body emotion interaction, *F*(4,92) = 44.434, *p* < .001, η^2^ = .659.

The proportion correct for sad faces varied as a function of body emotion, *F*(2,46) = 52.603, *p* < .001, η^2^ = .696. Participants were more accurate on congruent trials (*M* = .95) than on both incongruent sad face/fearful body trials (*M* = .79), *t*(23) = 9.407, *p* < .001, d = 1.92, and incongruent sad face/angry body trials (*M* = .92), *t*(23) = 2.907, *p* = .008, d = .59. Participants were more accurate on incongruent sad face/angry body trials (*M* = .92) than incongruent sad face/fearful body trials (*M* = .79), *t*(23) = 6.172, *p* < .001, d = 1.26. This pattern of results is inconsistent with both the dimensional theory and the emotional seed model; neither model predicts differential effects for fearful versus angry bodies on sad faces.

The proportion correct for fearful faces varied as a function of body emotion, *F*(2,46) = 39.173, *p* < .001, η^2^ = .630. Participants were more accurate on congruent trials (*M* = .84) than on both incongruent fearful face/sad body trials (*M* = .64), *t*(23) = 8.136, *p* < .001, d = 1.66, and incongruent fearful face/angry body trials (*M* = .70), *t*(23) = 6.029, *p* < .001, d = 1.23. Participants were more accurate on incongruent fearful face/angry body trials (*M* = .70) than incongruent fearful face/sad body trials (*M* = .64), *t*(23) = 2.669, *p* = .014, d = .545. This pattern of results is consistent with the emotional seed model, which predicts greater interference from sad bodies than angry bodies when judging fearful faces.

The proportion correct for angry faces varied as a function of body emotion, *F*(2,46) = 8.270, *p* = .001, η^2^ = .264. Participants were more accurate on congruent trials (*M* = .95) than on both incongruent angry face/fearful body trials (*M* = .87), *t*(23) = 3.567, *p* = .002, d = .73, and incongruent angry face/sad body trials (*M* = .90), *t*(23) = 3.442, *p* = .002, d = .70. Accuracy did not differ significantly across incongruent angry face/sad body and incongruent angry face/fearful body trials, *p* = .219. This pattern of results is inconsistent with both the dimensional theory and the emotional seed model; the dimensional theory predicts greater interference from fearful postures on angry faces whereas the emotional seed model predicts greater interference from sad postures.

#### Response Time

The main effect of face emotion, *F*(2,46) = 11.542, *p* < .001, η^2^ = .334, and the main effect of body emotion, *F*(2,46) = 5.283, *p* = .009, η^2^ = .187, showed that reaction times were slowest for fearful faces (all *p*s < .005) and fearful postures (all *p*s < .032). There was a significant face emotion x body emotion interaction, *F*(4,92) = 10.193, *p* < .001, η^2^ = .307 (Figure 3b). Congruency effects (longer response times on incongruent trials vs. congruent ones) were observed when angry faces were paired with fearful postures, *t*(23) = 2.78, *p* = .01, d = .57, but not sad postures, *p* = .08. Response times (RTs) were also slower when sad faces were paired with fearful postures than when sad faces were paired with angry postures, *t*(23) = 3.296, *p* = .003, d = .67, although both postures produced a significant increase in RTs. RTs for fearful faces did not differ when paired with angry versus sad bodies, *p* = .242, although both postures produced congruency effects when paired with fearful faces, *p*s < .05. This pattern of results is inconsistent with both the dimensional theory and the emotional seed model; the dimensional theory predicts stronger influence of fearful bodies on angry faces, but not sad faces, and the emotional seed model predicts stronger influence of fearful bodies on sad faces, but not on angry faces.

#### Errors Analysis

For trials on which sad faces were presented, fear was the target error. On congruent sad trials, the proportion of errors in which fear was selected (*M* = .52) did not differ from the .50 responding expected by chance, *t*(23) = .29, *p* = .774, indicating that there was no bias on congruent trials. As shown in Figure 3c, when viewing sad faces, the proportion of errors in which fear was selected differed across body postures, *F*(2,46) = 16.42, *p* < .001, η^2^ = .417. That proportion was significantly greater when a sad face was paired with a fearful body (*M* = .85) than when a sad face was paired with a sad body (*M* = .52), *F*(1,23) = 32.409, *p* < .001, η^2^ = .585; however, the proportion of errors in which fear was selected was not significantly different when a sad face was paired with an angry body (*M* = .43) than when a sad face was paired with a sad body (*M* = .52), *F*(1,23) = 1.298, *p* = .266, η^2^ = .053. In other words, presenting sad faces on angry bodies did not increase the proportion of errors in which anger was selected. This pattern of results is inconsistent with both models; neither model predicts that fearful bodies would be more influential than angry bodies when participants view sad faces.

For trials on which fearful faces were presented, sad was the target error. On congruent fear trials, the proportion of errors in which sad was selected (*M* = .82) was significantly different from chance, *t*(23) = 5.156, *p* < .001, indicating that participants were biased towards responding ‘sad’ even in the absence of conflicting information from body postures. The proportion of errors in which sad was selected did not differ across body postures, *F*(2,46) = 2.151, *p* = .128, η^2^ = .086. This pattern of results is partially consistent with the emotional seed model, which would predict a bias towards responding ‘sad’ versus ‘anger’ when viewing fearful faces; however, the proportion of errors in which sad was selected did not increase when fearful faces were presented on sad bodies relative to congruent trials.

For trials on which angry faces were presented, fear was the target error. On congruent trials, the proportion of errors in which fear was selected (*M* = .40) did not differ from chance, *t*(23) = -2.002, *p* = .057, indicating that there was no bias on congruent trials. The proportion of errors in which fear was selected varied across body posture, *F*(2,46) = 4.274, *p* = .02, η^2^ = .157. That proportion was significantly greater when an angry face was paired with a fearful body (*M* = .61) than when an angry face was paired with an angry body (*M* = .40), *F*(1,23) = 6.587, *p* = .017, η^2^ = .223. The proportion of errors in which fear was selected was not significantly different when an angry face was paired with a sad body (*M* = .40) than when an angry face was paired with an angry body (*M* = .40), *F*(1,23) = .001, *p* = .981. The greater impact of fearful bodies than sad bodies is consistent with the dimensional theory, but not the emotional seed model.

### Discussion

Participants showed robust congruency effects. For every facial expression, accuracy decreased when faces were presented on incongruent postures. In every case but one (angry faces presented on sad bodies), response times increased. These robust effects are consistent with previous studies [19,20,27,42] and confirm that adults’ perception of facial expressions is influenced by contextual cues. In two cases, incongruent body postures also shifted the pattern of errors; when sad and angry faces were presented on bodies posing fear, the proportion of fear errors increased relative to congruent trials.

The results of Experiment 1a lead to two significant conclusions. First, our results confirm that congruency effects are not always symmetrical, a pattern that is inconsistent with claims that the magnitude of congruency effects depends on similarity. Although accuracy on fear face trials was most impaired by sad postures and accuracy on sad face trials was most impaired by fear postures, symmetry was not observed in patterns of errors. Whereas fear postures shifted the pattern of errors (i.e., produced more ‘fear’ responses) on sad face trials, sad postures did not shift the pattern of errors on fear face trials. Furthermore, whereas fearful postures increased RTs on sad face trials more than angry postures, sad postures did not increase RTs on fear face trials more than angry postures. Likewise, angry postures increased RTs on sad face trials, but sad postures did not increase RTs on angry face trials, although for this pairing there was symmetry in effects on accuracy (which decreased) and error patterns (no change). Finally, whereas fearful postures shifted the pattern of errors on angry face trials, the reverse was not true.

Rather, our findings suggest that facial expressions and body postures may exert independent influences on the magnitude of congruency effects. Most notably, fearful postures were particularly influential. Fearful postures decreased accuracy and increased RTs on both sad-face trials and anger-face trials and were the only postures to shift the pattern of errors. Conversely, fearful faces may also be especially vulnerable. Relative to congruent trials, angry postures decreased accuracy by 14% for fearful faces but only 3% for sad faces; sad postures decreased accuracy by 20% for fearful faces but only 5% for angry faces.

Evidence that facial expressions and body postures exert independent influence on the magnitude of congruency effects is consistent with our second finding: Neither the dimensional theory nor the emotion seed model are entirely consistent with the pattern of results obtained. The dimensional theory would predict maximal influence when the two emotions presented in incongruent stimuli are fear and anger because fear and anger are both negatively valenced and high in arousal, whereas sad is negatively valenced but low in arousal [3,45]. In contrast, the emotional seed model predicts maximal effects when sad is displayed in either the face or the body of incongruent stimuli because sad faces are more physically similar to both fearful and angry faces than fearful and angry faces are to each other [39].

The dimensional theory was only consistent with one finding from our analyses of accuracy and response times: Response times to angry faces increased when angry faces were presented on fearful bodies but not sad bodies. However, accuracy for angry faces was not especially impaired by fearful bodies and neither accuracy for nor response times to fearful faces were especially impaired by angry bodies. In fact, accuracy for fearful faces was more impaired by sad bodies than angry bodies. Likewise, the emotional seed model is consistent with only one finding from our analyses of accuracy and response times: Accuracy for fearful faces was more impaired by sad bodies than by angry bodies. However, accuracy for angry faces was not more influenced by sad bodies than by fearful bodies and response times for angry faces increased more when angry faces were presented on fearful bodies than when they were presented on sad bodies. Notably, accuracy for and response times to sad faces were more impaired by fearful bodies than by angry bodies, a pattern that neither model predicts.

Our analyses of error patterns also failed to lend support to either model. On congruent trials a bias was observed for only one facial expression—fear; when making an error, participants were more likely to label fearful faces ‘sad’ than ‘anger’. The emotional seed model predicts this effect for sad faces (and these data are consistent with the pattern of errors observed in previous studies in which faces were presented in isolation [46,47]), but the complementary effects of sad faces being labeled ‘fear’ more than ‘angry’ and of angry faces being labeled ‘sad’ more than ‘fear’ were not observed. On incongruent trials, fearful bodies increased the proportion of ‘fear’ errors for both angry and sad faces. The dimensional theory predicts this effect for angry faces, but the complementary effect of angry bodies creating a bias for fearful faces was not observed and the dimensional theory does not predict that only fearful bodies would shift the pattern of errors for sad faces.

Our results are surprising given evidence from Aviezer, Hassin, Ryan, et al. [19] supporting the emotional seed model. They presented disgust faces in contexts depicting disgust, anger, sad, and fear. The magnitude of congruency effects was influenced by the physical similarity of facial displays of the emotion depicted by the context (which included but were not limited to body postures) and the facial expression of disgust. Congruency effects were largest for the angry context, smallest for the fearful context, and intermediate for the sad context. There are three potentially critical differences between our study and that of Aviezer, Hassin, Ryan, et al. [19]: 1) They only presented disgust faces, whereas we presented sad, anger, and fearful faces; 2) At least some of their contexts included props (e.g., a dirty diaper); and 3) They presented stimuli for an unlimited amount of time whereas our test stimuli were presented for 600ms. Varying both facial expressions and body postures was an essential component of our design and we intentionally limited contextual cues to body postures. However, to examine the influence of presentation times, we conducted Experiment 1b, which was identical to 1a except that stimuli were presented for an unlimited amount of time.

## Experiment 1b

### Method

#### Participants

Undergraduate students (*n* = 24) between the ages of 18 and 22 (*M* = 19.7) participated; they received partial course credit or a small monetary reward for their participation. All participants had normal or corrected-to-normal vision.

#### Materials and Procedure

The materials and procedure were identical to those used in Experiment 1a except that each stimulus was presented until a response was made. All participants met our criterion on isolated faces trials (*M* correct = .93). Every adult responded correctly on all catch trials and was very accurate on isolated body trials (*M* correct = .97).

### Results

#### Accuracy

**Figure 4:**
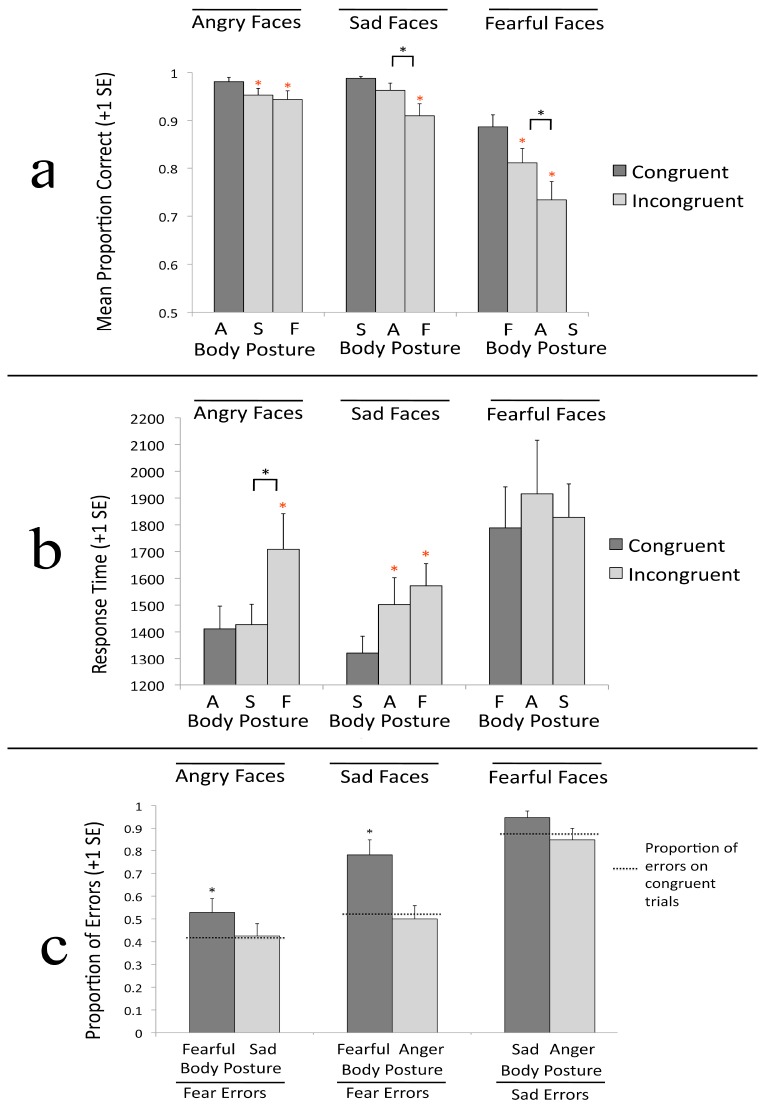
Results for Experiment 1b. a) Proportion of correct responses for each facial expression, presented by posture. b) Median reaction time results for each facial expression, presented by posture. Results are presented in milliseconds. c) Proportion of errors made for each facial expression, presented by posture. Dotted line represents the proportion of errors made by participants viewing congruent expressions, and was used as a baseline error level. * over individual bars indicate a significant difference between congruent and incongruent trials.

The 3 (face emotion: sad, fear, anger) x 3 (body emotion: sad, fear, anger) repeated measures ANOVA revealed a main effect of face emotion, *F*(2,46) = 26.073, *p* < .001, η^2^ = .531. As in Experiment 1a, accuracy was lower for fearful faces (*M* = .81) than for angry (*M* = .96), *t*(23) = -6.456, *p* < .001, d = 1.32, and sad faces (*M* = .95), *t*(23) = -4.639, *p* < .001, d = .95. Accuracy did not differ for sad versus angry faces, *p* = .647. The main effect for body emotion was marginally significant, *F*(2,46) = 3.093, *p* = .055, η^2^ = .119. Accuracy was lower for sad bodies (*M* = .89) than angry (*M* = .92), *t*(23) = -3.370, *p* = .003, d = .69, but not fearful bodies (*M* = .91), *t*(23) = -1.506, *p* = .146. Accuracy did not differ for angry versus fearful bodies, *p* = .670. As shown in Figure 4a, these main effects were qualified by a significant face emotion x body emotion interaction, *F*(4,92) = 19.404, *p* < .001, η^2^ = .458.

The proportion correct for sad faces varied as a function of body emotion, *F*(2,46) = 9.601, *p* < .001, η^2^ = .294. As in Experiment 1a, participants were more accurate on congruent trials (*M* = .99) than on incongruent sad face/fearful body trials (*M* = .91), *t*(23) = 3.392, *p* = .003, d = .69, but accuracy did not differ between the proportion correct on congruent trials versus incongruent sad face/angry body trials (*M* = .96), *p* = .06. As in Experiment 1a, participants were more accurate on incongruent sad face/angry body trials (*M* = .96) than incongruent sad face/fearful body trials (*M* = .91), *t*(23) = 3.046, *p* = .006, d = .62. This pattern of results is inconsistent with both the dimensional theory and the emotional seed model; neither model predicts differential effects of fearful versus angry bodies on perception of sad faces.

The proportion correct for fearful faces varied as a function of body emotion, *F*(2,46) = 20.388, *p* < .001, η^2^ = .470. As in Experiment 1a, participants were more accurate on congruent trials (*M* = .89) than on both incongruent fearful face/sad body trials (*M* = .73), *t*(23) = 5.10, *p* < .001, d = 1.04, and incongruent fearful face/angry body trials (*M* = .81), *t*(23) = 3.554, *p* = .002, d = .72. They were also more accurate on incongruent fearful face/angry body trials (*M* = .81) than incongruent fearful face/sad body trials (*M* = .73), *t*(23) = 4.039, *p* = .001, d = .82. As in Experiment 1a, this pattern of results is consistent with the emotional seed model; that model predicts greater interference from sad bodies than angry bodies when judging fearful faces.

The proportion correct for angry faces varied as a function of body emotion, *F*(2,46) = 3.227, *p* = .049, η^2^ = .123. As in Experiment 1a, participants were more accurate on congruent trials (*M* = .98) than both incongruent angry face/fearful body trials (*M* = .94), *t*(23) = 2.120, *p* = .045, d = .43, and incongruent angry face/sad body trials (*M* = .95), *t*(23) = 2.186, *p* = .039, d = .47. Accuracy did not differ across incongruent angry face/sad body trials and incongruent angry face/fearful body trials, *p* = .554. This pattern of results is inconsistent with both the dimensional theory and the emotional seed model; the dimensional theory predicts greater interference from fearful postures whereas the emotional seed model predicts greater interference from sad postures.

#### Response time

As in Experiment 1a, the main effect of face emotion, *F*(2,46) = 11.084, *p* < .001, η^2^ = .325, and the main effect of body emotion, *F*(2,46) = 5.330, *p* = .008, η^2^ = .188, indicate that response times were slowest for fearful faces (all *p*s < .004) and fearful bodies (all *p*s < .046). There was a significant face emotion x body emotion interaction, *F*(4,92) = 3.495, *p* = .011, η^2^ = .132. As shown in Figure 4b, in comparison to congruent expressions, only fearful postures increased reaction times to angry faces, *t*(23) = -3.755, *p* = .001, d = .77. For sad faces, both fearful, *t*(23) = 6.41, *p* < .001, d = 1.31, and angry, *t*(23) = 3.25, *p* = .004, d = .66, postures increased reaction times, F(2,46) = 13.437, p = .001, η^2^ = .369, but reaction times did not differ when sad faces were paired with fearful versus angry bodies, *p* = .202. No effects were observed for fearful faces, *p* = .597. These results provide only mixed support for both the dimensional theory and the emotional seed model.

#### Errors Analysis

The error analysis was conducted in the same manner as in Experiment 1a. For trials on which sad faces were presented, fear was the target error. As shown in Figure 4c, on congruent trials, the proportion of errors in which fear was selected (*M* = .52) did not differ from chance, *t*(23) = .44, *p* = .664, indicating there was no bias on congruent trials. When viewing sad faces, the proportion of errors in which fear was selected differed across body postures, *F*(2,46) = 8.754, *p* = .001, η^2^ = .276. That proportion was significantly greater when a sad face was paired with a fearful body (*M* = .78) than when a sad face was paired with a sad body (*M* = .52), *F*(1,23) = 11.754, *p* = .002, η^2^ = .338. The proportion of errors in which fear was selected was not significantly lower when a sad face was paired with an angry body (*M* = .50) than when a sad face was paired with a sad body (*M* = .52), *F*(1,23) = .089, *p* = .768, η^2^ = .004. This pattern of results is inconsistent with both the dimensional theory and the emotional seed model; neither model predicts that fearful bodies would be more influential than angry bodies when participants view sad faces.

For trials on which fearful faces were presented, sad was the target error. As in Experiment 1a, the proportion of errors in which sad was selected on congruent trials (*M* = .87) was significantly different than chance, *t*(23) = 8.263, *p* < .001, indicating that participants were biased towards responding ‘sad’ even in the absence of conflicting information from body postures. The proportion of errors in which sad was selected did not differ across body postures, *F*(2,46) = 1.813, *p* = .175, η^2^ = .073. As in Experiment 1a, this pattern of results is partially consistent with the emotional seed model, which would predict a bias towards ‘sad’ versus ‘anger’ errors when viewing fearful faces; however, the proportion of sad errors did not increase when fearful faces were presented on sad bodies relative to congruent trials.

For trials on which angry faces were presented, fear was the target error. On congruent trials, the proportion of errors in which fear was selected (*M* = .42) did not differ from chance, *t*(23) = -1.696, *p* = .103, indicating that there was no bias on congruent trials. Unlike Experiment 1a, the proportion of errors in which fear was selected did not differ across body posture, *F*(2,46) = 1.766, *p* = .182, η^2^ = .071. These results are inconsistent with both the dimensional theory and the emotional seed model. The dimensional theory predicts that fearful bodies will shift the pattern of errors most whereas the emotional seed model predicts sad bodies will shift the pattern of errors most.

### Discussion

As in Experiment 1a, participants showed robust congruency effects, although some effects observed in Experiment 1a did not replicate here. Despite unlimited presentation times, in every case but one (sad faces presented on angry bodies), accuracy decreased when faces were presented on incongruent postures. Not surprisingly, response times were less influenced by body postures when presentation times were unlimited; nonetheless, both fearful and angry bodies increased response times to sad faces and fearful bodies increased response times to angry faces. Fearful postures also increased the proportion of ‘fear’ errors in response to sad faces. No other effects were evident.

As in Experiment 1a, several asymmetries were evident in our results. Whereas sad postures impaired accuracy for angry faces, angry postures did not impair accuracy for sad faces. Whereas fear postures increased RTs to both fear and sad faces, neither sad nor angry bodies increased RTs to fearful faces. Finally, whereas fear postures shifted error patterns for sad faces, sad postures did not shift error patterns for fear faces. Given these asymmetries, it is not surprising that our results were not entirely consistent with either model of emotion perception. Most notably, although response times to angry faces increased when angry faces were presented on fearful bodies but not sad bodies, as predicted by dimensional theories, accuracy for angry faces was not especially impaired by fearful bodies and neither accuracy for nor response times to fearful faces were especially impaired by angry bodies. As in Experiment 1a, accuracy for fearful faces was more impaired by sad bodies than angry bodies. Likewise, although accuracy for fearful faces was more impaired by sad bodies than by angry bodies, as predicted by the emotional seed model, accuracy for angry faces was not more influenced by sad bodies than by fearful bodies and response times for angry faces increased more when angry faces were presented on fearful bodies than when they were presented on sad bodies. Our analyses of error patterns revealed a similar pattern to that observed in Experiment 1a and so failed to lend support to either model.

## Experiment 2

### Introduction

The results of Experiments 1a and 1b suggest that fearful postures have attentional privilege. Fearful postures impaired accurate perception of sad faces more than angry postures, they caused the largest increase in response times for both sad and angry faces (although the effect for angry faces was only marginally significant), and they were the only posture to alter the pattern of errors, causing participants to label the associated face as ‘fear’, rather than ‘sad’ or ‘angry’. What is unclear from Experiments 1a and 1b is whether the pervasive influence of fearful bodies reflects their ability to grab attention (see 43 for evidence that angry faces grab attention) across a wide range of conditions or reflects more specifically their influence on adults’ perception of emotional faces. To address this question we conducted a final experiment using a flanker task.

Flanker tasks have been widely used to examine selective attention. When a target stimulus (e.g., an *X*) is placed between two incongruent flankers (e.g., Os) adults’ response times increase relative to when the target is placed between congruent flankers (i.e., *X*s), despite being instructed to ignore the flanking stimuli [48]. Numerous stimulus characteristics influence the magnitude of this flanker effect including the spacing of stimuli [49,50], their size [51] and colour [52], and the presence/absence of motion [53]. Flanker effects for schematic faces are smaller for negatively valenced targets (e.g., sad) than positively valenced targets [54], consistent with a wealth of literature indicating that negative emotions narrow attention whereas positive emotions broaden attention ( [55–58] although see 59).

Based on this literature, we created a flanker task in which the body postures used in Experiment 1 served as stimuli. This task allowed us to determine whether postures depicting any one emotion preferentially capture and/or hold attention. This would be evident if flanker effects were especially small when postures depicting one emotion (e.g., fear) were presented as targets and especially large when postures depicting that same emotion were presented as flankers. Accuracy was expected to be high in all conditions (see 54,60–62), and so response time was our primary dependent variable.

#### Participants

Undergraduate students (*n* = 24) between the ages of 18 and 27 (*M* = 20.1) each received partial course credit or a small monetary reward for their participation.

#### Materials

**Figure 5:**
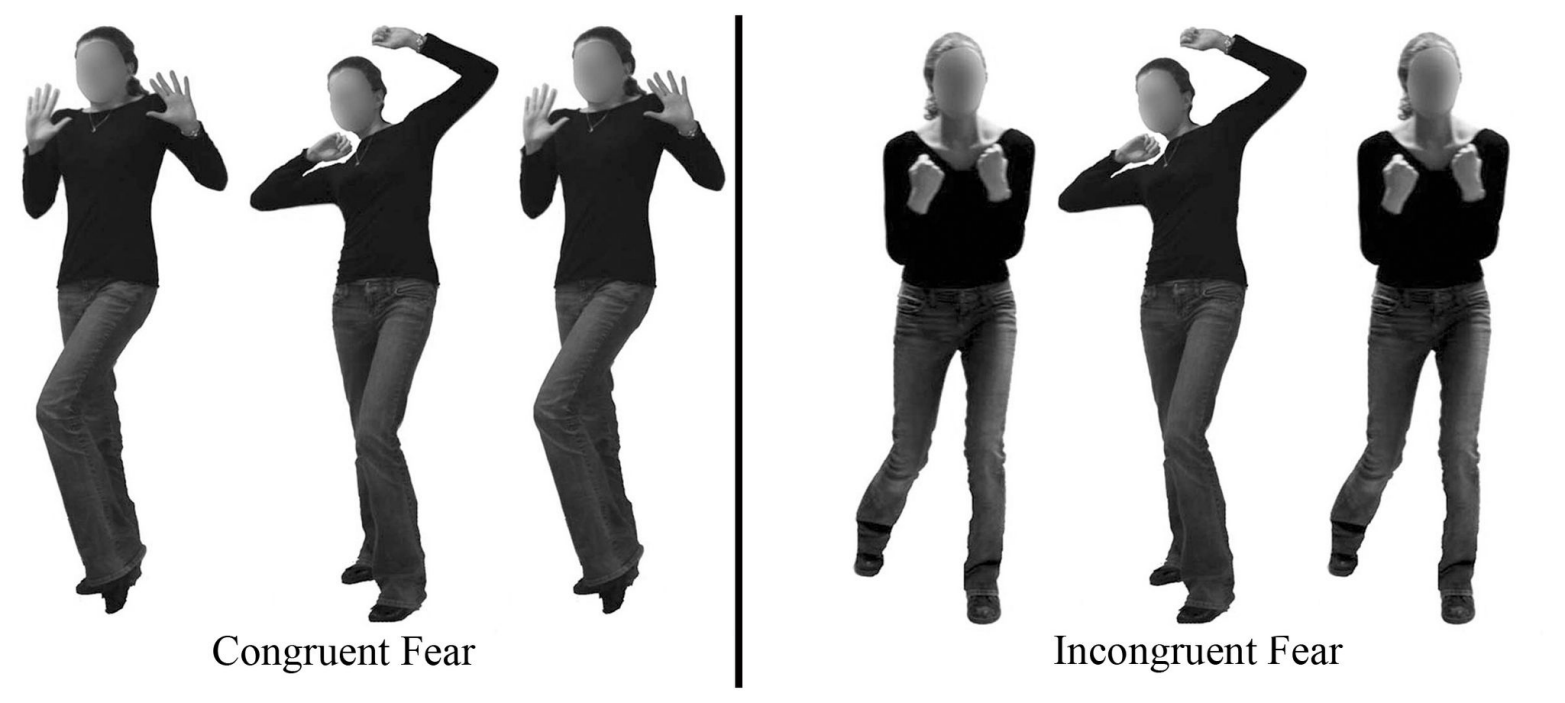
Stimuli presented in flanker task. Examples of Congruent (fearful body flanked by fearful bodies) and Incongruent (fearful body flanked by angry bodies) stimuli from Experiment 2.

Body postures were identical to those used in Experiments 1a and b except that the face on each stimulus was blurred out. Each stimulus array comprised a target body surrounded by two flanking bodies that were identical to each other; each posture served as the target for 5 arrays (1 congruent) and as flankers for 5 arrays (1 congruent). The inner edge of each flanking body was placed 1 cm away from the outermost edge of the target body. As shown in Figure 5, in each of the 24 congruent arrays the target posture was flanked by the alternative pose of the same emotion, conveyed by the same model as the target. In each of the 96 incongruent arrays, the target posture was flanked by postures posed by the same model but were conveying a different emotion than the target; each target was paired with each of the four possible flankers (2 emotions x 2 poses).

#### Procedure

The procedure began with a practice block comprised of 36 trials (12 congruent). Participants were instructed to ignore the flankers and indicate as quickly as possible which of three emotions was depicted by the target body. Practice trials were designed to familiarize participants with the procedure and were immediately followed by 288 test trials, divided into four blocks of 72. (Each practice trial was repeated as a test trial.) The 24 congruent displays and 48 of the incongruent displays were presented in each block. Within each block, each target posture was presented twice on incongruent trials—once with each of the two incongruent emotions being depicted as flankers. Stimuli were presented until a response was made. The trials within each block were presented in a different random order to each participant.

### Results

#### Accuracy

A 3 (target emotion: sad, fear, anger) x 3 (flanker emotion: sad, fear, anger) repeated measures ANOVA was conducted to determine whether the proportion correct differed across trials. Although accuracy was high (≥ 95%) for each of the three body emotions, there was a main effect for target emotion, *F*(2,46) = 4.101, *p* = .023, η^2^ = .151. Accuracy was higher on sad target trials (*M* = .98) than on anger target trials (*M* = .95), *t*(23) = 2.205, *p* = .038, d = .45, but not fear target trials (*M* = .98), *t*(23) = .332, *p* = .743. Accuracy was marginally higher on fear target trials than anger target trials, *t*(23) = 2.037, *p* = .053, d = .42. Notably, there was no main effect for flanker emotion, *p* = .576, and no target emotion x flanker emotion interaction, *p* = .524.

#### Response times

A 3 (target emotion: sad, fear, anger) x 3 (flanker emotion: sad, fear, anger) repeated measures ANOVA was conducted to determine whether response times differed across trials. Neither the main effects nor the interaction were significant, all ps > .10.

### Discussion

With the exception of accuracy being slightly lower when angry bodies served as targets (although still high at 95%), neither accuracy nor response times varied across emotions. In short, in the absence of a task that required identification of facial expressions, flanking body postures did not interfere with emotion perception and it was not the case that postures associated with any one emotion (i.e., fear) enjoyed attentional privilege.

The lack of flanker effect cannot be attributed to our task being too easy; flanker effects have been observed in several previous studies in which accuracy approached ceiling [54,60–62]. In addition, our finding accurate recognition of all body postures ensures that differential congruency effects in Experiments 1a and 1b cannot be attributed to some postures being hard to recognize. The lack of flanker effect also cannot be attributed to the perception of body postures being impervious to contextual influences. Prior research has shown that body postures are more accurately recognized when presented in a scene depicting the same versus a different or neutral emotion [63], adults’ ability to recognize emotions in point-light displays was impaired for some (e.g., love, joy) emotions but enhanced for others (e.g., sadness, fear) when only one member of an interacting dyad was shown [9], and in a match-to-sample task performance in matching body postures was impaired when the distracter was an angry posture compared to when the distracter was a happy, sad, or fearful posture [64]. Rather, the results of Experiment 2 suggest that complex patterns of congruency effects in which fearful postures are more influential than sad and angry postures are not universal and may be limited to situations in which participants judge facial expressions. A future study in which faces are targets and bodies serve as flankers (and vice versa) would provide further insights about the limitations of congruency effects.

## General Discussion

The overarching goal of the current studies was to deepen our understanding of the mechanisms underlying the influence of contextual cues – specifically postural context – on adults’ perception of facial displays of emotion. Although some have argued that incongruent stimuli like those presented in the current experiments are irrelevant because they are seen infrequently in the real world [21], this argument has not been supported with empirical evidence [22] and does not consider that these stimuli provide a useful tool for furthering our understanding of the *processes underlying* the perception and categorization of facial expressions. Previous studies have shown that body posture influences the accuracy with which participants categorize facial expressions and that the magnitude of this congruency effect depends on the similarity of the emotion displayed in the face and the emotion displayed in the context [19,42]. Furthermore, these effects occur rapidly (i.e., are evident in P100 [20]), occur automatically and without effort [65], and likely reflect holistic processing [26,42]. Thus, the use of incongruent stimuli has provided evidence that would have remained undiscovered using traditional stimuli.

The results of Experiments 1a and 1b did not provide overwhelming support for either the emotional seed model or the dimensional theory in explaining congruency effects. Sad bodies did not systematically interfere more with participants’ perception of fearful and angry faces as predicted by the emotional seed model, nor did fearful and angry bodies systematically interfere more with angry and fearful faces, respectively, as predicted by the dimensional theory. Instead, asymmetries were abundant and in particular fearful bodies were associated with pervasive congruency effects (see introduction to Experiment 2 for summary). This result is intriguing because fearful bodies did not influence participants’ responses in all situations; in Experiment 2, the fearful flankers were no more likely to cause flanker effects than other emotional flankers. These data suggest that it is only in the context of a face/body pairing that fearful bodies are overly influential.

One explanation for the pervasive effects associated with fear postures is the relatively high level of errors associated with categorizing fearful facial expressions ( [16,66,67]; see also 15). Detecting fear in conspecifics is important; fear alerts the observer to potential danger, allowing an appropriate and timely response (e.g., flight/fight). To the extent that fear is difficult to recognize in facial expressions, being able to detect fear from distal cues (e.g., voices, body movements) is advantageous. It is possible, then, that fear postures are especially influential for this reason; they may be heavily relied upon when detecting fear in the real world.

Our results suggest that both dimensional theories and the emotional seed model need to be refined if they are to account for and predict congruency effects. In terms of the dimensional theory, one modification that would allow the theory to explain our results would be the addition of a third dimension. Dimensions such as dominance/submissiveness [68] or attention/rejection [37] have been suggested previously. Gao et al. [47] used multidimensional scaling based on similarity ratings to show that three- or four-dimensional structures were optimal for adults. The dimensions represented were pleasure (or valence), dominance (strong vs. weak), arousal (high vs. low), and intensity. Anger is on one end of the dominance dimension, representing feelings of power, dominance, and impulses to act, whereas fear is on the other end, representing feelings of weakness, submission, and inaction. This is consistent with participants viewing angry postures as displaying approach tendencies and fearful postures as displaying avoidance tendencies, even when presented with incongruent facial expressions [69]. Thus, taking dominance into account would increase the similarity between fear and sadness, thereby explaining the large congruency effects found in our data when sad and fear were paired. Although adding a third dimension may cause both models to make more similar predictions for the emotions of anger, fear, and sadness, the underlying mechanisms of these predictions would remain unique and could therefore be tested in different ways.

Another potential refinement to the dimensional theory would be to take into account the weighting given to each dimension when determining emotional state. We hypothesize that judgments of emotion may be influenced most by perceived valence. Sensitivity to valence emerges early in development [36,38,45,70] and in the multidimensional scaling study by Gao et al. [47] valence was the primary dimension. Greater weighting of the valence dimension also makes adaptive sense; it is probably most important to know whether someone else is feeling positive or negative than it is to know whether they are highly aroused (although of course both dimensions are informative). Although this refinement to the dimensional theory would not alter our interpretation of the data presented here, heavier weighting of the valence dimension may explain evidence that happy faces are protected from congruency effects when paired with sad, fearful, and angry bodies ( [32,42,71,72]; see also catch trials in Experiments 1a and b in which happy faces were presented on congruent and incongruent bodies). Varying the relative weights of the underlying dimensions would alter the shape of the circumplex, thereby changing similarities among expressions.

The main point of the emotional seed model is that similarity in physical characteristics (akin to action units described by Ekman and Friesen [40]) will influence error patterns when labeling isolated faces and the magnitude of congruency effects when faces are placed in incongruent contexts. Based on multidimensional scaling studies by Susskind et al. [39], Aviezer and colleagues [13,19] predicted that the influence of context on adults’ perception of disgust faces would decrease as the distance between emotions on the MDS plots increased. However the seed model does not address asymmetries of influence: a disgust face is influenced by an anger body more than an anger face is influenced by a disgust body [26]. In addition, although data from both human subjects and machine learning based on a set of training stimuli indicate that sad lies between and equidistant from fear and anger faces (see Figure 6 in [39]), we note that when subsequently tested on a new set of face stimuli there was more noise in the circumplex. In particular, the MDS plots varied across individual participants with some plots (see Figure 7 in [39]) showing fear in closer proximity to anger than sad, and others showing fear in closer proximity to sad than anger. Future studies should collect similarity data (or a confusion matrix) for individual participants and for individual exemplars in order to provide a more powerful test of the hypothesis that the magnitude of congruency effects will vary as a function of similarity of the emotion in the face and body. Fearful bodies may interfere most with perception of sad faces for individuals who judge sad faces to be most similar to fear faces and for specific exemplars of sad faces that are easily confused with fear. Considering asymmetries in influence and individual variations in expression perception would generate a more nuanced model of the mechanisms underlying congruency effects.

Ultimately, we believe it is important to note that these models do not need to be in conflict. Neither model considers the source of the expressions presented, but it is possible that similarities in expressions – for example, fear and surprise – are caused by similarities in arousal or valence. Therefore, future research in which these models can be reconciled may provide the greatest advances in our understanding of the mechanisms underlying emotional expression processing.

### Summary

We have reported that congruency effects for face-body stimuli are restricted to adults’ perception of facial expressions and vary across emotions displayed in the face and in the body — the strongest effects are observed for sad and angry faces paired with fearful bodies. The nature of congruency effects is more complex than indicated by early research and needs further examination. Body postures have a powerful influence on both adults and children’s [42,71] perception of facial displays of emotion; given that face-body stimuli have more ecological validity than isolated faces, future research investigating the mechanisms underlying emotion perception and its development should include contextual cues. 
